# Food allergy-induced anaphylaxis impairs adrenal adrenergic biosynthesis despite preserved vagal afferent–adrenal sympathetic reflex function

**DOI:** 10.1016/j.jphyss.2026.100097

**Published:** 2026-09-06

**Authors:** Marina Khatun, Nishat Akther, Sajjad Hossain, Sakurako Asano, Risa Shimane, Nanaka Tameike, Kakeru Shimizu, Yukari Takeda, Mamoru Tanida, Yusaku Iwasaki, Yuta Masuda, Chikara Abe

**Affiliations:** a Department of Physiology, University of Fukui Faculty of Medical Sciences, Fukui, Japan; b Department of Physiology II, Kanazawa Medical University, Ishikawa, Japan; c Laboratory of Animal Science, Graduate School of Life and Environmental Sciences, Kyoto Prefectural University, Kyoto, Japan; d Department of Biochemistry and Molecular Biology, Faculty of Life Science, Mawlana Bhashani Science and Technology University, Bangladesh

**Keywords:** Anaphylaxis, Food allergy, Adrenaline, Vagal afferent nerve, Adrenal sympathetic nerve

## Abstract

Food-induced anaphylaxis is a life-threatening allergic reaction in which endogenous sympathoadrenal responses play a critical role in maintaining physiological homeostasis. However, the functional status of autonomic pathways regulating adrenal catecholamine output during anaphylaxis remains unclear. Using a mouse model of food allergy-induced anaphylaxis, we examined the vagal afferent–medullary C1 neuron–adrenal sympathetic pathway and adrenal adrenergic function. Ovalbumin challenge induced hypotension, hypothermia, metabolic acidosis, and elevated plasma catecholamines. Despite severe systemic deterioration, vagal afferent stimulation and optogenetic activation of C1 neurons continued to evoke robust adrenal sympathetic responses, indicating preserved autonomic regulation. In contrast, adrenal transcriptomic analysis revealed enrichment of hypoxia-related pathways and selective suppression of phenylethanolamine N-methyltransferase (PNMT), a key enzyme for adrenaline synthesis. Oxygen supplementation partially restored PNMT expression, increased plasma adrenaline levels, and improved physiological outcomes. These findings identify hypoxia-associated adrenal dysfunction as a potential limitation of endogenous adrenergic compensation during anaphylactic shock.

## 1 Introduction

Food-induced anaphylaxis refers to a serious systemic allergic reaction triggered by ingestion of specific allergens, typically with rapid onset and potentially life-threatening outcomes [1]. Upon exposure to food allergens, IgE antibodies bound to mast cells become cross-linked, leading to mast cell activation. This process initiates the release of preformed inflammatory mediators such as histamine, tryptase, carboxypeptidase A, and proteoglycans, as well as newly synthesized substances including leukotrienes, tumor necrosis factor-α (TNF-α), and platelet-activating factor [2]. These mediators collectively contribute to the classical features of anaphylaxis—vasodilation, angioedema, bronchoconstriction, and enhanced mucous secretion. Among available treatments, adrenaline (epinephrine) remains the gold standard for managing severe anaphylactic reactions [3], [4]. Its therapeutic efficacy stems from its broad pharmacological profile: α₁-adrenergic receptor stimulation causes vasoconstriction and elevates blood pressure; β₁-adrenergic receptor stimulation increases cardiac contractility; and β₂-adrenergic receptor activation leads to bronchodilation and suppression of further inflammatory mediator release [5]. Both experimental animal studies and clinical experience consistently demonstrate that intramuscular adrenaline administration rapidly mitigates life-threatening symptoms [1], [6]. In human cases, delayed administration of adrenaline is a major risk factor associated with fatal anaphylaxis.

The adrenal medulla plays a pivotal role in systemic homeostasis under stress, primarily through the secretion of catecholamines. Chromaffin cells in the adrenal medulla store and release a mixture of adrenaline (∼80%), noradrenaline (∼16%), and dopamine (∼4%) [7]. These catecholamines are synthesized sequentially from tyrosine, with adrenaline formed via conversion of noradrenaline by phenylethanolamine N-methyltransferase (PNMT). Secretion is tightly regulated by cholinergic (acetylcholine-mediated) stimulation, which induces calcium-dependent exocytosis of secretory granules. Thus, efficient catecholamine biosynthesis and secretion are essential for adaptive responses to hypotension and airway constriction during anaphylaxis.

Despite the existence of this robust endogenous defense system, severe anaphylaxis still frequently requires exogenous adrenaline administration, suggesting that endogenous adrenergic compensation may become insufficient during systemic allergic reactions. However, the mechanisms underlying this potential failure remain unclear. One possibility is that autonomic neural pathways regulating adrenal function become impaired during anaphylaxis. In this regard, we focused on medullary C1 neurons, a specialized population of glutamatergic/catecholaminergic/peptidergic neurons located in the rostral ventrolateral medulla (RVLM) [8]. These neurons are highly responsive to physiological stressors such as hypotension and hypercapnia and exert regulatory control over autonomic and neuroendocrine responses [9]. Our previous work demonstrated that activation of C1 neurons robustly enhances adrenal sympathetic nerve activity (AdSNA), and that selective stimulation of cervical vagal afferents subsequently activates C1 neurons [9], [10], [11]. These findings suggest that the vagal afferent–C1 neuron–adrenal sympathetic pathway may constitute an important endogenous defense circuit during anaphylactic stress.

Alternatively, the adrenal medulla itself may become functionally impaired during anaphylaxis, even if autonomic neural signaling remains preserved. In such a scenario, adrenal catecholamine biosynthesis or secretion may fail to adequately compensate for the severe systemic stress induced by allergic reactions. However, whether food allergy-induced anaphylaxis disrupts autonomic neural regulation, adrenal medullary function, or both has not been elucidated.

In the present study, we investigated whether endogenous adrenergic defense during food allergy-induced anaphylaxis is impaired at the level of autonomic neural circuitry or adrenal medullary function. To address this question, we examined systemic physiological responses, adrenal sympathetic nerve activity, vagal afferent- and C1 neuron-evoked adrenal responses, adrenal catecholamine output, and adrenal gene expression during anaphylaxis. Clarifying the mechanisms underlying endogenous adrenergic dysfunction may provide important insights into the pathophysiology of anaphylactic shock and identify novel therapeutic strategies for severe food allergy.

## 2 Material and methods

### 2.1 Animals

All animal procedures were conducted in accordance with the Guiding Principles for the Care and Use of Animals in the Field of Physiological Science established by the Physiological Society of Japan. The experimental protocols were approved by the Animal Research Committees of the University of Fukui (R08013) and Gifu University (AG-P-N-20230026). OVA-IgE/Jcl mice were obtained from CLEA Japan, Inc. and maintained as heterozygous on a BALB/c genetic background. Age-matched BALB/c littermates lacking the OVA-specific IgE transgene were used as negative controls. A total of 191 male (n = 130) and female (n = 61) mice, aged 8–20 weeks, were used in the experiments. Food allergy-induced anaphylaxis was triggered by intravenous injection of ovalbumin (OVA; 40 mg/kg, i.v.; A5503, Sigma-Aldrich) in OVA-IgE/Jcl mice. Bovine serum albumin (BSA; 40 mg/kg, i.v.; 10738328103, Roche) was used as a control antigen.

### 2.2 Anesthesia and postoperative management

All surgical procedures were conducted under aseptic conditions. For most surgeries, mice were anesthetized via intraperitoneal injection of a ketamine (120 mg/kg) and xylazine (12 mg/kg) mixture. Anesthetic depth was confirmed by the absence of corneal and hind-paw withdrawal reflexes. Supplemental doses (10% of the original dose, i.p.) were administered as needed to maintain surgical anesthesia. Body temperature was maintained at 37.0 ± 0.5 °C using a servo-controlled heating pad throughout the procedure. Upon completion of surgery, mice received subcutaneous injections of atipamezole (an α₂-adrenergic antagonist; 2 mg/kg), penicillin G potassium (3000 U/kg), and ketoprofen (4 mg/kg) for postoperative care. Animals were housed in groups of four per cage under a 12:12-hour light–dark cycle, with ambient temperature maintained at 24 ± 1 °C.

For recordings of adrenal sympathetic nerve activity (AdSNA), mice were anesthetized with urethane (500 mg/kg, i.p.). Anesthetic depth was monitored as described above, and supplemental urethane (10% of the initial dose, i.p.) was administered as needed to maintain stable anesthesia throughout the recording period. Body temperature was maintained at 37.0 ± 0.5 °C using a servo-controlled heating pad.

### 2.3 Whole-body plethysmography and respiratory gas analysis

Respiratory measurements were conducted in conscious, unrestrained mice using a whole-body plethysmography system (Data Sciences International). The plethysmography chamber was continuously flushed with dry air at a flow rate of 1.0 standard liter per minute (Alicat Scientific, USA) and maintained at room temperature. Respiratory signals were digitized at a sampling rate of 1 kHz using an analog-to-digital converter (PowerLab, ADInstruments). Prior to data collection, mice were habituated to the chamber environment for 6 h. To evaluate respiratory responses during food allergy-induced anaphylaxis, respiratory rate and respiratory waveform amplitude were continuously recorded following intravenous administration of ovalbumin (OVA) or bovine serum albumin (BSA).

### 2.4 Arterial pressure and heart rate measurement in conscious mice

To monitor cardiovascular responses during food allergy-induced anaphylaxis, mice were implanted with a radio-telemetry probe (PA-C10; Data Sciences International) at least two weeks before physiological recordings. Under aseptic conditions, the catheter tip was inserted into the right common carotid artery. Arterial pressure (AP) and heart rate (HR) were continuously recorded using a PhysioTel receiver system (RLA1020; Data Sciences International). Mean AP and HR were derived from pulsatile arterial pressure waveforms using calibration values obtained during transmitter implantation.

### 2.5 Measurement of body temperature and locomotor activity

Body temperature (BT) and locomotor activity were monitored using an implantable programmable device (NanoTag®; Kissei Comtec Co., Ltd.). Under aseptic conditions, the device was surgically implanted into the abdominal cavity. After a one-week recovery period, the device was programmed to record BT and activity at 5-minute intervals via a non-contact integrated circuit (IC) card system. At the end of the experiment, recorded data were wirelessly retrieved using the same IC card reader.

### 2.6 Blood sampling and measurement of plasma humoral factors

Blood samples were collected from the ophthalmic artery using heparinized glass capillary tubes (Fisherbrand Microhematocrit Capillary Tubes; Fisher Scientific) under 2% isoflurane anesthesia. Adequate anesthetic depth was confirmed by the absence of corneal and hind-paw withdrawal reflexes. Blood gas parameters, including partial pressure of carbon dioxide (PCO₂), pH, bicarbonate (HCO₃⁻), and hematocrit, were immediately measured using an electrolyte and blood gas analyzer (IDEXX Laboratories). Plasma samples were then obtained by centrifugation at 4000 rpm for 10 min.

For catecholamine analysis, plasma samples were mixed with 10% sodium disulfite (final concentration: 2%) and the internal standard 3,4-dihydroxybenzylamine (5 pmol). Catecholamines were purified using activated alumina and quantified by high-performance liquid chromatography with electrochemical detection (HPLC-ECD; HTEC-500, EICOM).

### 2.7 Electrical stimulation of vagal afferents

The left cervical vagus nerve was exposed through a midline cervical incision and carefully separated from surrounding connective tissue. A bipolar silver wire electrode (AS633; Cooner Wire) was placed around the nerve for electrical stimulation. To selectively activate vagal afferent fibers, the nerve was transected and electrical stimulation was delivered to the central (cranial) cut end. Electrical stimulation of the vagal afferent nerve consisted of monophasic square-wave pulses (50 μA, 0.1 ms pulse duration). Single pulses were delivered at 1-s intervals, and a total of 120 stimuli were applied for each recording trial.

### 2.8 Optogenetic activation of RVLM neurons

An adeno-associated viral vector encoding channelrhodopsin-2 fused to mCherry under the control of the CaMKIIα promoter (AAV-CaMKIIa-hChR2(H134R)-mCherry) was injected into the rostral ventrolateral medulla (RVLM). The viral vector was obtained from the UNC Vector Core (https://www.med.unc.edu/genetherapy/vectorcore/).

Mice were anesthetized with ketamine (120 mg/kg, i.p.) and xylazine (12 mg/kg, i.p.). A small incision was made to expose the mandibular branch of the facial nerve for subsequent electrical stimulation used to localize the facial motor nucleus (FN). Animals were then placed in a stereotaxic apparatus (SR-6M-HT, Narishige). A glass micropipette (inner diameter: 1.2 mm; tip diameter: ∼25 μm) filled with viral solution was inserted through a 1.5-mm craniotomy positioned caudal to the parieto-occipital suture. To identify the RVLM, the facial nerve was electrically stimulated (0.1 ms pulse duration, 1–300 μA, 1 Hz), and antidromic field potentials were recorded from the FN through the virus-containing pipette. The RVLM was identified based on its location caudal to the FN. Three injections of viral solution (140 nL each) were delivered 300 μm above the ventral medullary surface, with injection sites spaced 200 μm apart along the rostrocaudal axis. Following viral delivery, an optical fiber (125 μm core diameter after desheathing; numerical aperture 0.39; Thorlabs) was implanted 300 μm above the injection sites. The optical fiber was mounted in a zirconia ferrule (outer diameter: 1.25 mm; bore diameter: 130 μm; Precision Fiber Products) and secured to the skull using dental cement.

Optogenetic stimulation consisted of blue light pulses (10 mW, 10 ms pulse duration) delivered at 1-s intervals. High-frequency 10-s photostimulation (20 Hz, 10 ms pulse duration) was additionally applied to evaluate the magnitude of AdSNA activation.

### 2.9 Nerve recordings

The adrenal sympathetic nerve was carefully isolated and placed onto bipolar stainless-steel electrodes (AS633; Cooner Wire) while maintaining nerve integrity. The nerve-electrode interface was embedded in silicone elastomer (Kwik-Sil; World Precision Instruments) to stabilize the recording and reduce movement artifacts. Neural signals were amplified using a differential amplifier (AB-611J, Nihon Kohden) with a band-pass filter set at 150–1000 Hz. The amplified signals were processed through a gate circuit to minimize baseline noise and rectified using an absolute-value circuit. Processed signals were digitized at 1000 Hz with an analog-to-digital converter (PowerLab, ADInstruments).

To evaluate stimulus-evoked adrenal sympathetic nerve activity (AdSNA), peristimulus waveform averages were generated from half-wave rectified signals. Averaged responses were constructed from 120 sweeps during vagal afferent stimulation and 180 sweeps during optogenetic photostimulation. High-frequency photostimulation (20 Hz) was additionally applied to evaluate the magnitude of AdSNA activation. Sham stimulation was performed by setting stimulation intensity to 0 μA for electrical stimulation or 0 mW for optical stimulation. At the end of each experiment, mice were euthanized with an overdose of ketamine and xylazine (3 × surgical dose, i.p.), and complete disappearance of nerve activity was confirmed.

### 2.10 Immunohistochemistry

Mice were deeply anesthetized with an overdose of pentobarbital sodium and transcardially perfused with 50 mL of heparinized saline, followed by 100 mL of freshly prepared 4% paraformaldehyde in 100 mM sodium phosphate buffer (pH 7.4). Brains were removed, post-fixed in the same fixative at 4 °C for 48 h, and sectioned coronally at 40 μm thickness using a cryotome. Sections were stored at −20 °C in cryoprotectant solution containing 20% glycerol and 30% ethylene glycol in 50 mM phosphate buffer (pH 7.4). To verify viral expression, free-floating sections were processed for immunofluorescence staining. mCherry was detected using a goat polyclonal anti-RFP antibody (1:1000; 200–101–379, Rockland), followed by a donkey anti-goat Alexa Fluor 594-conjugated secondary antibody (1:200; Jackson ImmunoResearch Laboratories). Tyrosine hydroxylase (TH)-positive neurons were identified using a rabbit polyclonal anti-TH antibody (1:1000; AB152, Millipore), followed by a donkey anti-rabbit Alexa Fluor 488-conjugated secondary antibody (1:200; Jackson ImmunoResearch Laboratories). Fluorescent images were obtained using a BZ-X800 fluorescence microscope (KEYENCE). Brightness and contrast were adjusted uniformly to preserve all signal-containing pixels without altering the original data. No additional image manipulation was performed.

### 2.11 Subdiaphragmatic vagal afferent and adrenal sympathetic denervation

For selective subdiaphragmatic vagal afferent denervation, a midline laparotomy was performed to expose the esophagus and subdiaphragmatic vagal trunks. A freshly prepared capsaicin solution (0.1% w/v in 10% ethanol, 10% Tween-80, and 80% saline) was applied to the anterior and posterior vagal trunks using a fine brush for 30 min. Care was taken to avoid contact with surrounding tissues. Following treatment, the surgical area was thoroughly rinsed with sterile saline and the abdominal wall was closed in layers. Mice recovered on a heating pad after surgery. Capsaicin was applied locally without systemic administration to minimize off-target effects. Sham-operated mice underwent the same procedure but received vehicle solution only.

For bilateral adrenal sympathetic denervation, a small flank incision was made to expose the adrenal gland by gentle kidney retraction. Under a stereomicroscope, the adrenal sympathetic nerve running along the adrenal vasculature was carefully identified and transected using fine iridectomy scissors while preserving surrounding vessels and connective tissue. The same procedure was performed bilaterally. Incisions were closed with absorbable sutures and surgical adhesive. Sham-operated animals underwent identical surgical exposure without nerve transection.

### 2.12 RNA-sequencing library preparation and sequencing

Adrenal glands were harvested 60 min after intravenous administration of OVA or BSA, immediately snap-frozen in liquid nitrogen, and stored at −80°C until RNA extraction. RNA quality was assessed using the RNA 6000 Pico Kit (5067–1513, Agilent Technologies, Waldbronn, Germany). Sequencing libraries were prepared from 3.5 μL of total RNA using the SMARTer Stranded Total RNA-Seq Kit v2 - Pico Input Mammalian (635005, Takara Bio, Mountain View, CA, USA) according to the manufacturer’s protocol at half-volume scale. To optimize amplification conditions, a 1 μL aliquot of the pre-amplified library was subjected to quantitative real-time PCR using Fast SYBR Green Master Mix (4385612, Applied Biosystems, Thermo Fisher Scientific, Foster City, CA, USA). The optimal PCR cycle number was determined based on template abundance, and index primers were incorporated during the final amplification step. Amplified libraries were purified and eluted in 16 μL of a 1:1 mixture of Stranded Elution Buffer and nuclease-free water.

Additional RNA-seq libraries were generated using the NEBNext rRNA Depletion Kit (Human/Mouse/Rat) and the NEBNext Ultra II Directional RNA Library Prep Kit for Illumina (E6310 and E7420, New England Biolabs, Ipswich, MA, USA). Final PCR products were purified using the QIAquick PCR Purification Kit (28106, Qiagen, Hilden, Germany) and eluted in EB buffer. Library quality and concentration were evaluated using the High Sensitivity DNA Kit (5067–4626, Agilent Technologies). Libraries were pooled at equimolar concentrations and subjected to paired-end sequencing (36 bp) on a NextSeq 500 platform (Illumina, San Diego, CA, USA) using the High Output Kit v2 (75 cycles, FC-404–2005) at the Tsukuba i-Laboratory (Tsukuba, Japan). FASTQ files were generated using the BaseSpace Onsite platform.

### 2.13 Sequencing data analysis

FASTQ files were imported into CLC Main Workbench (CLC-MW, version 24.0.1; Qiagen). Reads were mapped to the mouse reference genome (mm10) and quantified using a 49,585-gene annotation set downloaded from the CLC-MW server. Gene expression levels were calculated as Total Count and reads per kilobase of transcript per million mapped reads (RPKM). Heatmap generation, gene clustering, pathway enrichment analysis, and Gene Ontology analysis were performed using RNAseqChef (https://imeg-ku.shinyapps.io/RNAseqChef/) [12] with default analysis parameters.

### 2.14 Quantitative analysis of adrenal mRNA expression

Total RNA was extracted from adrenal glands using the NucleoSpin RNA Plus kit (Takara Bio). RNA concentration and purity were assessed spectrophotometrically based on the 260/280 nm absorbance ratio. Reverse transcription and quantitative real-time PCR were performed using the One Step TB Green PrimeScript PLUS RT-PCR Kit (Takara Bio) according to the manufacturer’s instructions. Glyceraldehyde-3-phosphate dehydrogenase (GAPDH) was used as the internal control gene. Relative mRNA expression levels were calculated using the 2 −ΔΔCt method. Primer sequences are listed in the Supplementary Table.

### 2.15 Statistical analysis

All statistical analyses were performed using GraphPad Prism version 11 (GraphPad Software, San Diego, CA, USA). All datasets were tested for normality using either the D’Agostino–Pearson omnibus normality test or the Kolmogorov–Smirnov test. Homogeneity of variance was assessed using the Brown–Forsythe test. When assumptions of normality and equal variance were satisfied, statistical comparisons were performed using either unpaired *t*-test, one-way ANOVA, or two-way ANOVA, followed by Dunnett’s, Bonferroni’s, or Tukey’s post hoc multiple-comparison tests when appropriate. Data are presented as mean ± standard error of the mean (SEM). Statistical significance was defined as P < 0.05.

## 3 Results

### 3.1 Anaphylaxis induces transient respiratory alterations and sustained cardiovascular collapse in conscious mice

**Fig. 1:**
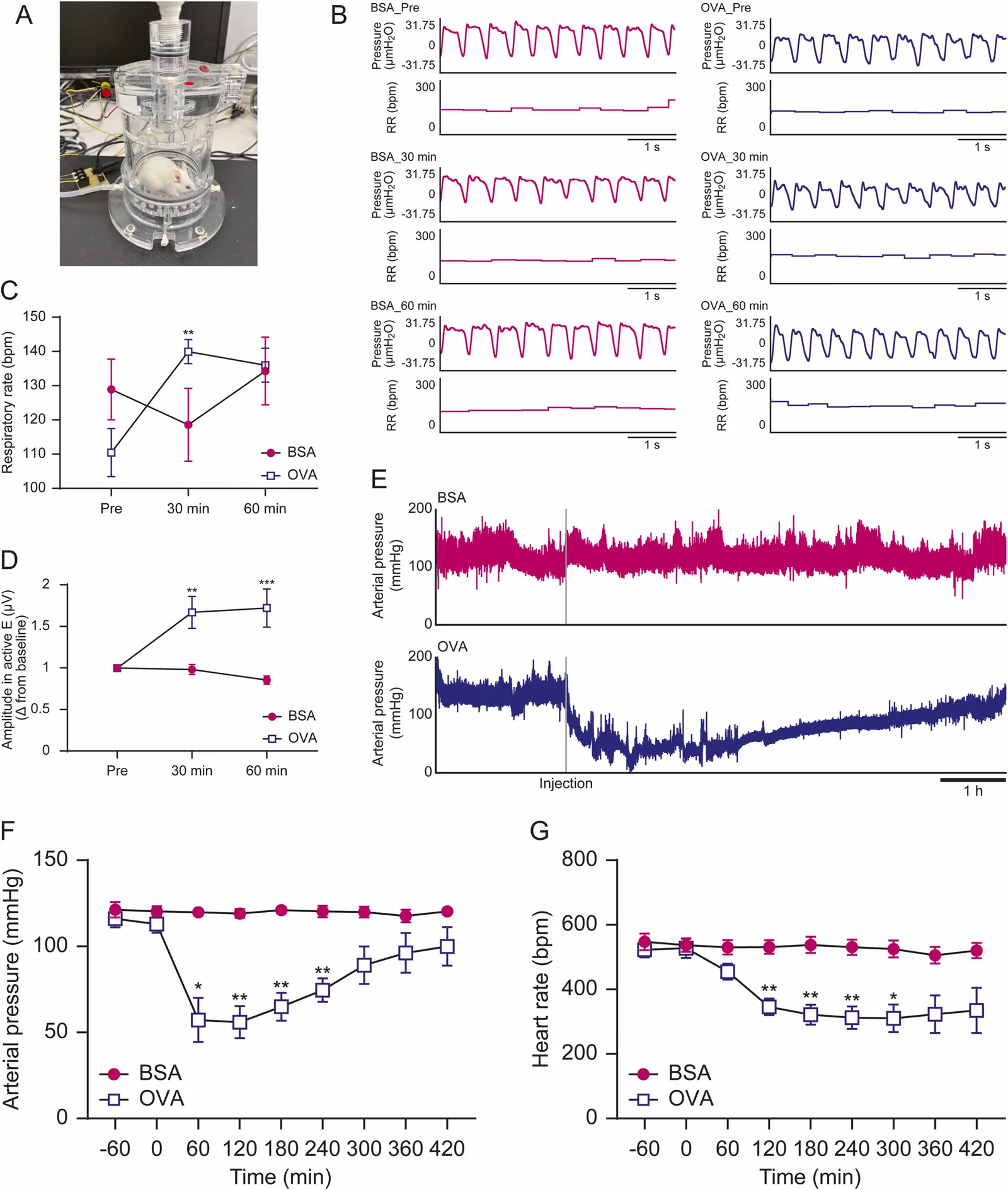
Anaphylaxis induces transient respiratory alterations and sustained cardiovascular collapse in conscious mice (A) Whole-body plethysmography chamber used for respiratory recordings in conscious, unrestrained mice. (B) Representative respiratory traces obtained from OVA-treated (magenta, left) and BSA-treated (blue, right) mice at baseline (Pre), 30 min, and 60 min after injection. (C-D) Summary data showing respiratory rate (C) and expiratory pressure amplitude (Δ from baseline; D) before (Pre), 30 min, and 60 min after injection (n = 6 per group). Data are presented as mean ± SEM. **P < 0.01 and ***P < 0.001 versus Pre by Dunnett’s multiple comparisons test. (E) Representative arterial pressure (AP) traces recorded before and after OVA injection. (F-G) Time-course changes in mean arterial pressure (F) and heart rate (G) (n = 6 per group). Data are presented as mean ± SEM. *P < 0.05 and **P < 0.01 versus Pre by Dunnett’s multiple comparisons test.

We first characterized the systemic physiological responses to food allergy-induced anaphylaxis in conscious mice by examining respiratory and cardiovascular parameters. Respiratory activity was evaluated using whole-body plethysmography (Fig. 1A). Representative respiratory traces demonstrated a marked increase in respiratory rate (RR) and respiratory waveform amplitude 30 min after OVA administration compared with baseline recordings (Fig. 1B).

Quantitative analysis revealed a significant increase in RR at 30 min following OVA injection, which returned to near baseline levels by 60 min (Fig. 1C). No significant changes were observed in BSA-treated control mice. In parallel, expiratory pressure waveform amplitude was significantly elevated after OVA administration (Fig. 1D).

Cardiovascular responses were continuously monitored using radiotelemetry. OVA challenge induced a profound and sustained reduction in arterial pressure (AP) that persisted for at least 240 min after injection (Figs. 1E and 1F). Heart rate (HR) was also significantly reduced following OVA administration (Fig. 1G), consistent with severe systemic cardiovascular dysregulation during anaphylactic shock. These changes were not observed in BSA-treated mice.

### 3.2 Thermoregulatory and metabolic alterations during food allergy-induced anaphylaxis

**Fig. 2:**
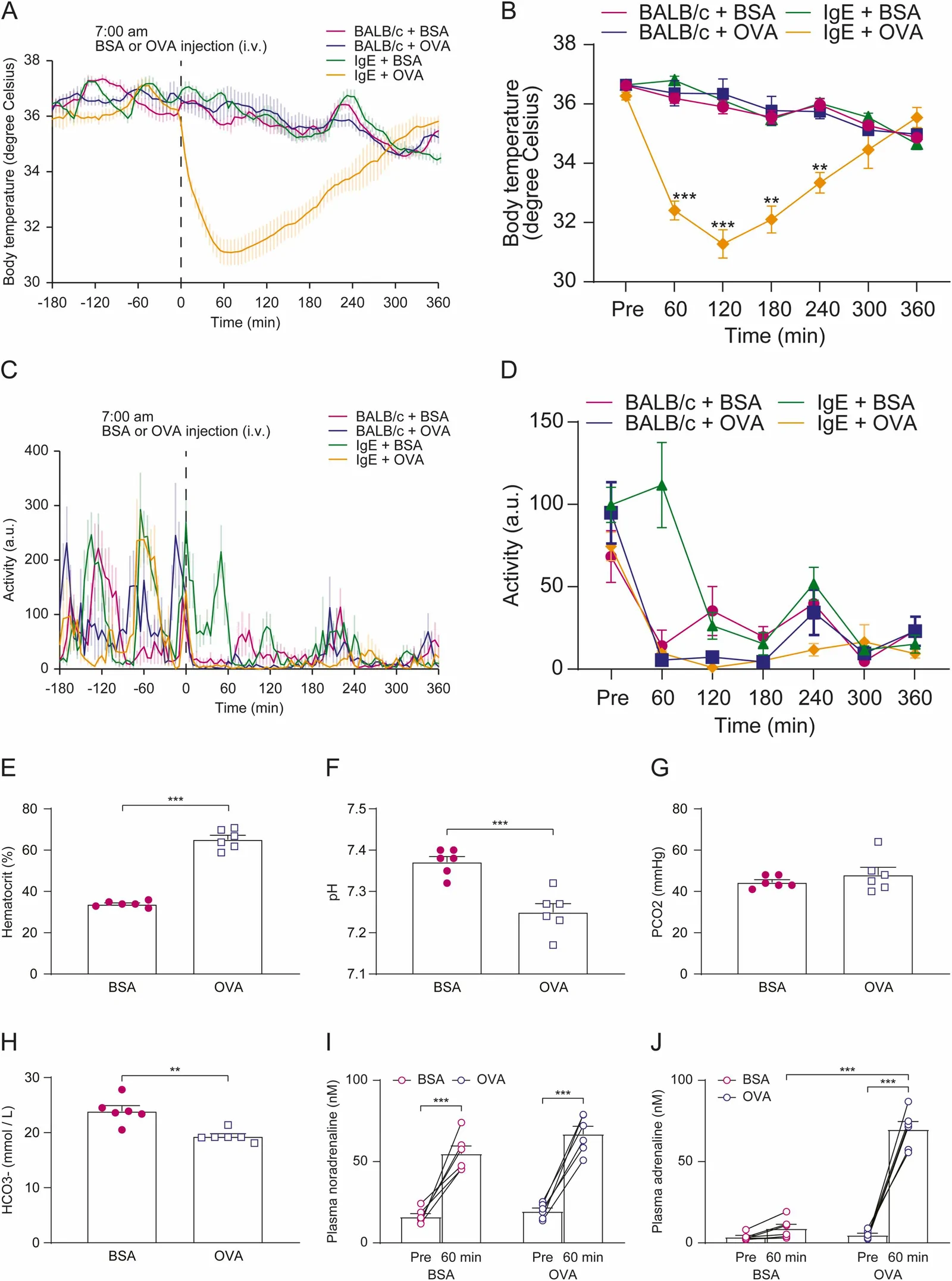
Thermoregulatory and metabolic alterations during food allergy-induced anaphylaxis (A) Time-course of body temperature (BT) measured using implanted telemetry devices in BALB/c and IgE-sensitized mice following intravenous injection of OVA or BSA. (B) Quantitative analyses of BT at each time point (n = 6 per group). Data are presented as mean ± SEM. **P < 0.01 and ***P < 0.001 versus Pre by Dunnett’s multiple comparisons test. (C) Time-course of locomotor activity measured in BALB/c and IgE-sensitized mice following intravenous injection of OVA or BSA. (D) Quantitative analyses of locomotor activity at each time point (n = 6 per group). (E-H) Quantitative analysis of hematocrit (E), blood pH (F), blood PCO₂ (G), and bicarbonate (HCO₃⁻) levels (H) measured 60 min after injection (n = 6 per group). Data are presented as mean ± SEM. ***P < 0.001 by unpaired *t*-test. (I-J) Quantitative analysis of plasma noradrenaline (I) and adrenaline (J) concentrations measured before (Pre) and 60 min after injection (n = 6 per group). Data are presented as mean ± SEM. **P < 0.01 and ***P < 0.001 by Bonferroni’s multiple comparisons test.

We next examined thermoregulatory and metabolic responses during food allergy-induced anaphylaxis by monitoring body temperature, locomotor activity, and blood biochemical parameters following OVA administration. IgE-sensitized mice challenged with OVA exhibited a profound and sustained decrease in body temperature that developed shortly after antigen administration and persisted for at least 240 min (Figs. 2A and 2B). In contrast, control groups (BALB/c + BSA, BALB/c + OVA, and IgE + BSA) maintained stable body temperature throughout the recording period. Locomotor activity was also monitored continuously. Although OVA-challenged mice exhibited severe hypothermia, overall locomotor activity was not significantly suppressed compared with control groups during the observation period (Figs. 2C and 2D).

To further characterize systemic physiological alterations during anaphylaxis, blood samples were collected 60 min after antigen challenge. OVA-treated mice showed a significant increase in hematocrit levels (Fig. 2E), indicating marked hemoconcentration. Blood pH was significantly reduced in OVA-challenged mice (Fig. 2F), whereas partial pressure of CO₂ (PCO₂) remained unchanged (Fig. 2G). In parallel, plasma bicarbonate (HCO₃⁻) levels were significantly decreased (Fig. 2H), indicating the development of metabolic acidosis during anaphylaxis. Despite severe systemic physiological collapse, plasma catecholamine concentrations were markedly elevated following OVA administration. Both plasma noradrenaline and adrenaline levels were significantly increased 60 min after antigen challenge (Figs. 2I and 2J).

### 3.3 Electrically evoked vagal-adrenal responses are preserved during anaphylaxis

**Fig. 3:**
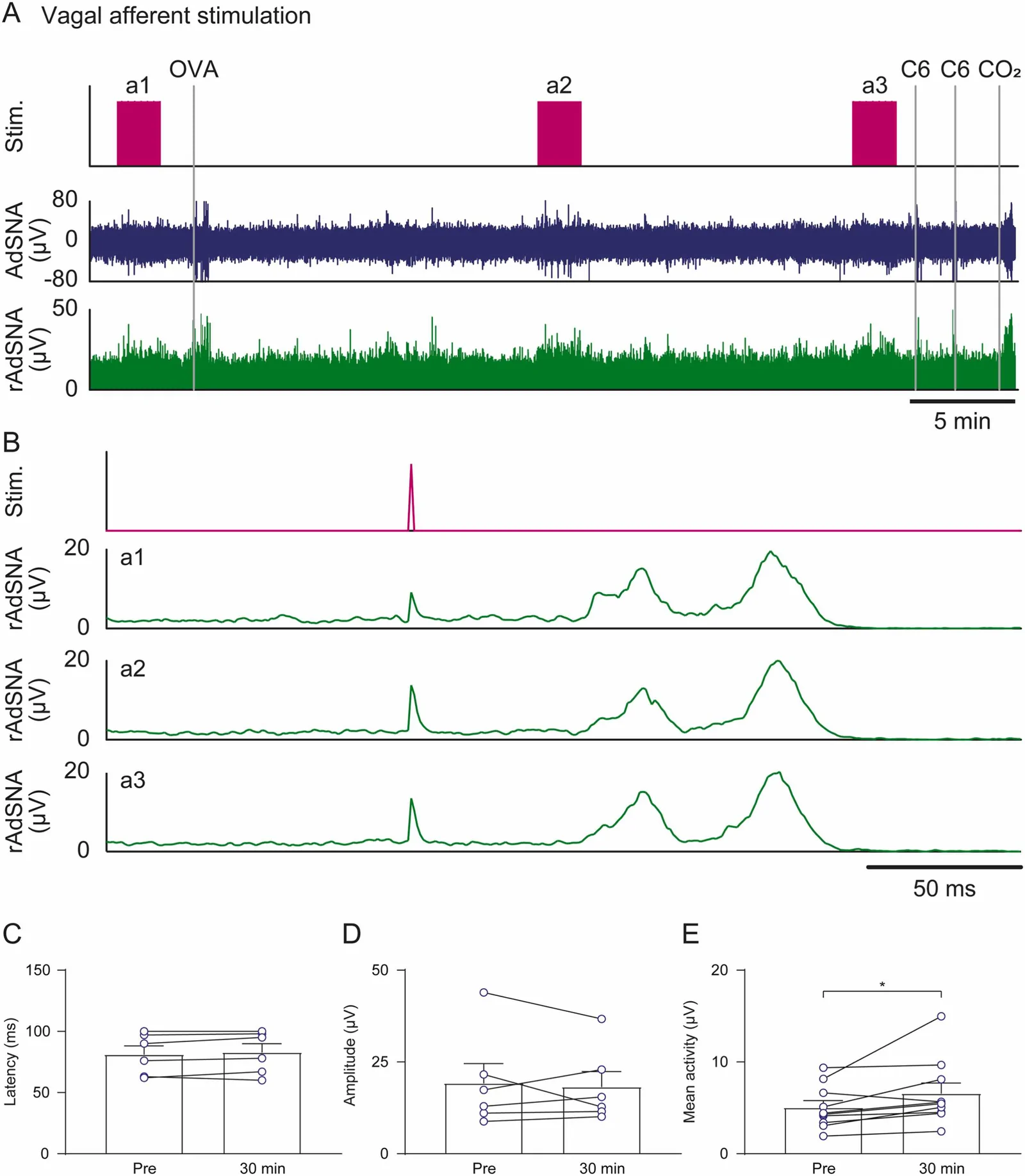
Electrically evoked vagal-adrenal responses are preserved during systemic anaphylaxis (A) Representative traces of raw adrenal sympathetic nerve activity (AdSNA) and rectified AdSNA (rAdSNA) recorded in anesthetized mice during selective electrical stimulation of vagal afferent fibers (50 μA, 120 sweeps per trial). Three stimulation trials were performed: a1 (Pre), a2 (15 min after OVA injection), and a3 (30 min after OVA injection). (B) Averaged rAdSNA waveforms aligned to stimulus onset for each trial (a1–a3). (C-D) Quantitative analyses of response latency (C) and peak amplitude (D) of evoked adrenal sympathetic responses before and after OVA administration (n = 6). (E) Mean baseline rAdSNA calculated over a 1-minute period prior to stimulation of the vagal afferent nerve (n = 10). Data are presented as mean ± SEM. *P < 0.05 by unpaired *t*-test.

To determine whether food allergy-induced anaphylaxis disrupts autonomic neural regulation of the adrenal gland, we recorded adrenal sympathetic nerve activity (AdSNA) in response to selective electrical stimulation of vagal afferent fibers. Electrical stimulation of the cervical vagal afferent nerve (50 μA, 120 sweeps) reliably evoked short-latency adrenal sympathetic responses both before and after OVA administration (Figs. 3A and 3B). Three stimulation sessions were performed: prior to OVA injection (a1), 15 min after OVA injection (a2), and 30 min after OVA injection (a3). Reflex AdSNA responses remained clearly detectable throughout the anaphylactic period. Notably, AdSNA persisted even after administration of the ganglionic blocker hexamethonium (C6), while hypercapnic stimulation (CO₂ challenge) induced robust increases in AdSNA, consistent with our previous reports [9].

Quantitative analysis demonstrated that neither the latency nor the peak amplitude of the evoked responses was significantly altered following OVA administration (Figs. 3C and 3D), indicating preservation of vagal afferent-mediated autonomic signaling during anaphylaxis. In contrast, baseline rectified AdSNA (rAdSNA) measured over a 1-minute recording period was significantly increased 30 min after OVA injection (Fig. 3E).

### 3.4 C1 neuron-mediated adrenal sympathetic responses are preserved during anaphylaxis

**Fig. 4:**
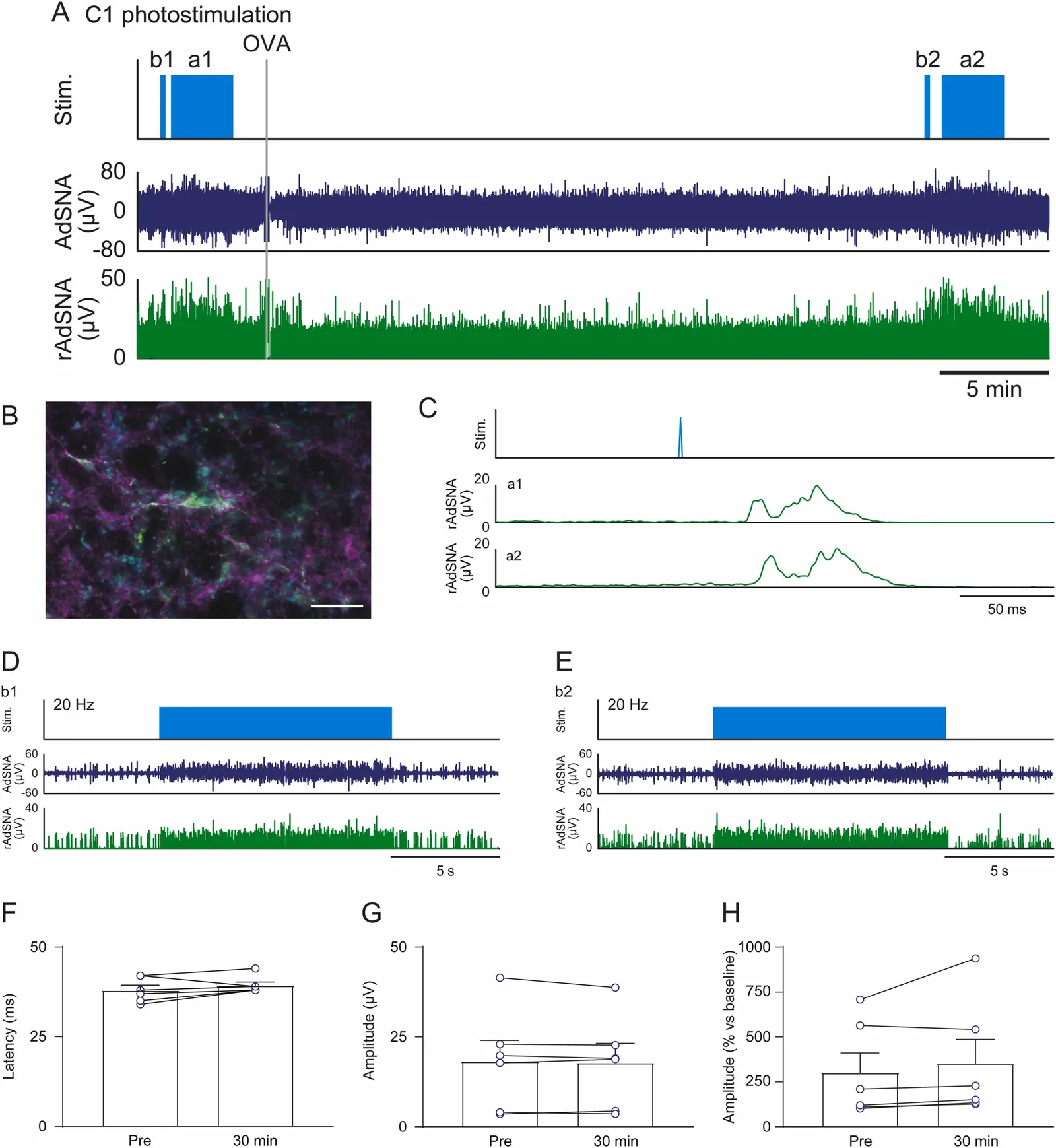
C1 neuron-mediated adrenal sympathetic responses are preserved during anaphylaxis (A) Representative traces of raw adrenal sympathetic nerve activity (AdSNA) and rectified AdSNA (rAdSNA) recorded in anesthetized mice during selective optogenetic stimulation of medullary C1 neurons. Photostimulation (10 mW, 180 sweeps) was applied before OVA injection (a1) and 30 min after OVA injection (a2). High-frequency photostimulation trials (20 Hz; b1 and b2) were additionally performed immediately before each stimulation session. (B) Representative fluorescence image showing ChR2-mCherry expression (magenta) and tyrosine hydroxylase (TH)-positive neurons (green) within the RVLM. Scale bar = 100 μm. (C) Averaged rAdSNA waveforms aligned to photostimulation onset during a1 and a2 trials. (D-E) Representative traces of raw AdSNA and rAdSNA during continuous high-frequency (20 Hz) photostimulation at b1 (Pre) and b2 (30 min after OVA injection). (F-G) Quantitative analyses of response latency (F) and peak amplitude (G) of C1 neuron-evoked adrenal sympathetic responses before and after OVA administration (n = 6). (H) Quantification of high-frequency photostimulation-induced AdSNA responses expressed as percentage increase from baseline (n = 6). Data are presented as mean ± SEM.

To further determine whether central autonomic circuitry remains functional during food allergy-induced anaphylaxis, we examined adrenal sympathetic nerve activity (AdSNA) evoked by selective optogenetic stimulation of RVLM C1 neurons. Photostimulation of C1 neurons (10 mW, 180 sweeps) reliably elicited adrenal sympathetic responses both before and after OVA administration (Fig. 4A–C). Two stimulation sessions were performed: before OVA injection (a1) and 30 min after OVA injection (a2). C1 neuron photostimulation continued to evoke clear adrenal sympathetic responses during anaphylaxis. Quantitative analysis demonstrated that neither the latency nor the peak amplitude of the evoked responses was significantly altered following OVA administration (Figs. 4F and 4G).

To further evaluate pathway excitability, high-frequency photostimulation (20 Hz) was applied before (b1) and 30 min after OVA administration (b2) (Figs. 4D and 4E). The magnitude of AdSNA activation induced by high-frequency stimulation did not differ significantly between pre- and post-anaphylactic conditions (Fig. 4H).

### 3.5 Role of vagal afferents and adrenal sympathetic nerves in systemic responses to anaphylaxis

**Fig. 5:**
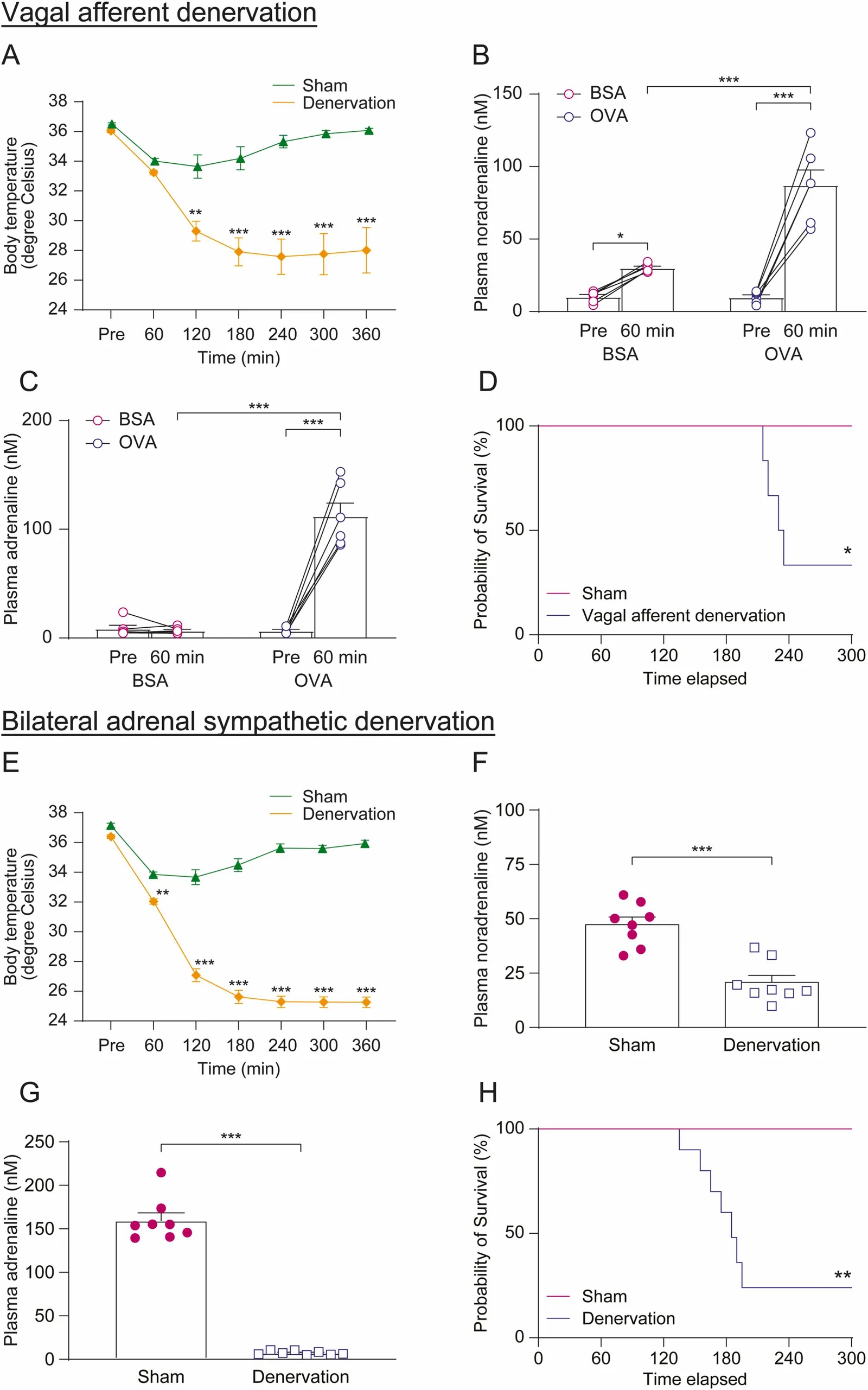
Vagal afferent signaling and adrenal sympathetic innervation contribute to systemic homeostasis during anaphylaxis (A) Time-course changes in body temperature following OVA administration in sham- operated (n = 6) and vagal afferent denervated (n = 6) mice. Data are presented as mean ± SEM. **P < 0.01 and ***P < 0.001 versus sham-operated mice by Bonferroni’s multiple comparisons test. (B-C) Quantitative analysis of plasma noradrenaline (B) and adrenaline (C) concentrations measured before (Pre) and 60 min after BSA or OVA injection in vagal afferent denervated mice. Data are presented as mean ± SEM. *P < 0.05 and ***P < 0.001 by Bonferroni’s multiple comparisons test. (D) Survival probability in sham-operated and vagal afferent denervated mice following OVA challenge (n = 6 per group). *P < 0.05 by Log-rank (Mantel–Cox) test. (E) Time-course changes in body temperature following OVA administration in sham-operated (n = 6) and adrenal sympathetic denervated (n = 8) mice. Data are presented as mean ± SEM. **P < 0.01 and ***P < 0.001 versus sham-operated mice by Bonferroni’s multiple comparisons test. (F-G) Quantitative analysis of plasma noradrenaline (F) and adrenaline (G) concentrations measured 60 min after OVA injection in sham-operated (n = 8) and adrenal sympathetic denervated (n = 8) mice. Data are presented as mean ± SEM. ***P < 0.001 by unpaired *t*-test. (H) Survival probability in sham-operated and adrenal sympathetic denervated mice following OVA challenge (n = 6 per group). **P < 0.01 by Log-rank (Mantel–Cox) test.

To determine the contribution of vagal afferent signaling to systemic homeostatic regulation during food allergy-induced anaphylaxis, we performed selective subdiaphragmatic vagal afferent denervation. Following OVA administration, vagal afferent denervated mice developed a marked and sustained hypothermic response, with body temperature significantly decreased from 60 min after antigen challenge and remaining suppressed throughout the 6-hour observation period compared with the sham- operated mice (Fig. 5A). Despite disruption of vagal afferent signaling, plasma catecholamine levels remained significantly elevated following OVA administration (Fig. 5B and C). However, survival analysis demonstrated that vagal afferent denervated mice exhibited significantly lower survival rates compared with sham-operated controls after OVA challenge (Fig. 5D).

We next evaluated the role of adrenal sympathetic innervation by performing bilateral adrenal sympathetic denervation. Similar to vagal afferent denervated mice, adrenal-denervated animals exhibited profound and prolonged hypothermia after OVA administration compared with the sham-operated mice (Fig. 5E). In contrast to vagal afferent denervated mice, adrenal sympathetic denervation markedly suppressed plasma catecholamine responses during anaphylaxis. Plasma noradrenaline and adrenaline concentrations were significantly reduced 60 min after OVA injection compared with sham-operated controls (Fig. 5F and G). Consistent with impaired sympathoadrenal compensation, adrenal-denervated mice also exhibited significantly reduced survival following OVA challenge (Fig. 5H).

### 3.6 Adrenal transcriptomic remodeling reveals impaired adrenaline biosynthetic machinery during anaphylaxis

**Fig. 6:**
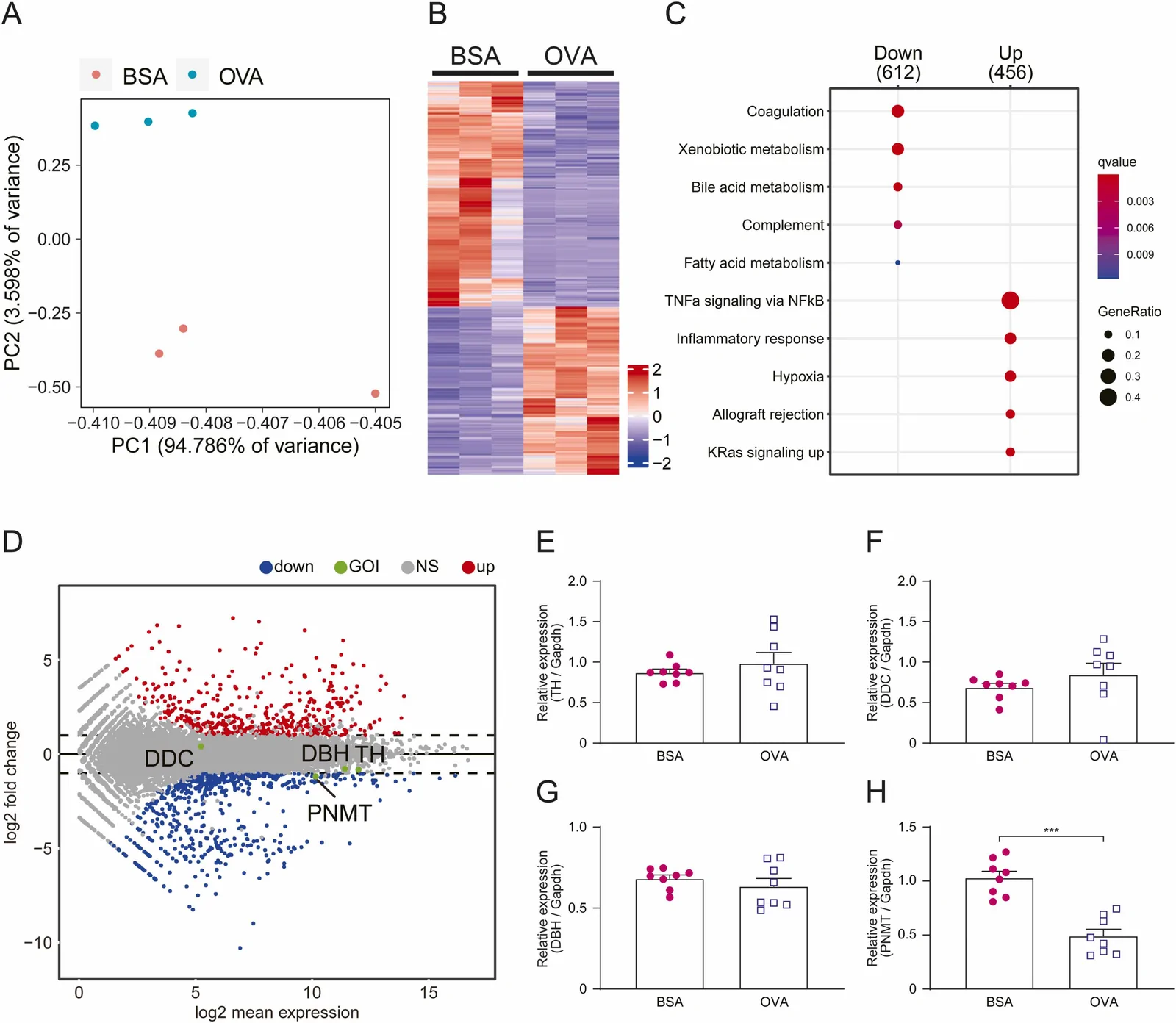
Adrenal transcriptomic remodeling reveals impaired adrenaline biosynthetic machinery during anaphylaxis (A) Principal component analysis (PCA) of adrenal RNA-seq datasets obtained from OVA-challenged (n = 3) and BSA-treated control (n = 3) mice. (B) Heatmap of differentially expressed genes (DEGs) showing widespread transcriptional remodeling in the adrenal gland following OVA challenge. A total of 612 genes were downregulated and 456 genes were upregulated. (C) Pathway enrichment analysis using MSigDB Hallmark gene sets, demonstrating enrichment of inflammatory, coagulation, and hypoxia-related pathways. (D) MA plot showing log2 fold change versus mean expression. Genes related to catecholamine biosynthesis are highlighted, including tyrosine hydroxylase (TH), dopa decarboxylase (DDC), dopamine β-hydroxylase (DBH), and phenylethanolamine N-methyltransferase (PNMT). (E-H) Quantitative PCR validation of catecholamine biosynthetic enzyme expression, including TH (E), DDC (F), DBH (G), and PNMT (H) (n = 8 per group). Data are presented as mean ± SEM. ***P < 0.001 by unpaired *t*-test.

To investigate molecular alterations in the adrenal gland during food allergy-induced anaphylaxis, we performed RNA-sequencing (RNA-seq) analysis of adrenal tissues collected 60 min after OVA administration (Supplementary table). Principal component analysis (PCA) demonstrated clear separation between OVA-challenged and BSA-treated control groups, indicating substantial transcriptional remodeling in the adrenal gland during anaphylaxis (Fig. 6A). Consistent with this finding, heatmap analysis identified widespread changes in gene expression, including 612 downregulated genes and 456 upregulated genes in OVA-treated mice (Fig. 6B). Pathway enrichment analysis using MSigDB Hallmark gene sets revealed significant enrichment of pathways related to TNF-α signaling via NF-κB, inflammatory responses, coagulation, and hypoxia (Fig. 6C), suggesting that adrenal tissues undergo marked inflammatory and hypoxic stress during systemic anaphylaxis.

We next focused on genes involved in catecholamine biosynthesis. MA plot analysis highlighted the expression profiles of tyrosine hydroxylase (TH), dopa decarboxylase (DDC), dopamine β-hydroxylase (DBH), and phenylethanolamine N-methyltransferase (PNMT), which collectively mediate adrenaline synthesis in adrenal chromaffin cells (Fig. 6D). Among these genes, PNMT, the enzyme responsible for converting noradrenaline to adrenaline, was selectively downregulated following OVA challenge. To validate the RNA-seq findings, quantitative PCR analysis was performed for TH, DDC, DBH, and PNMT. Whereas TH, DDC, and DBH expression levels were not significantly altered (Fig. 6E–G), PNMT expression was significantly reduced in OVA-treated mice compared with BSA controls (Fig. 6H).

### 3.7 Oxygen supplementation partially restores adrenal adrenaline biosynthesis and systemic homeostasis during anaphylaxis

**Fig. 7:**
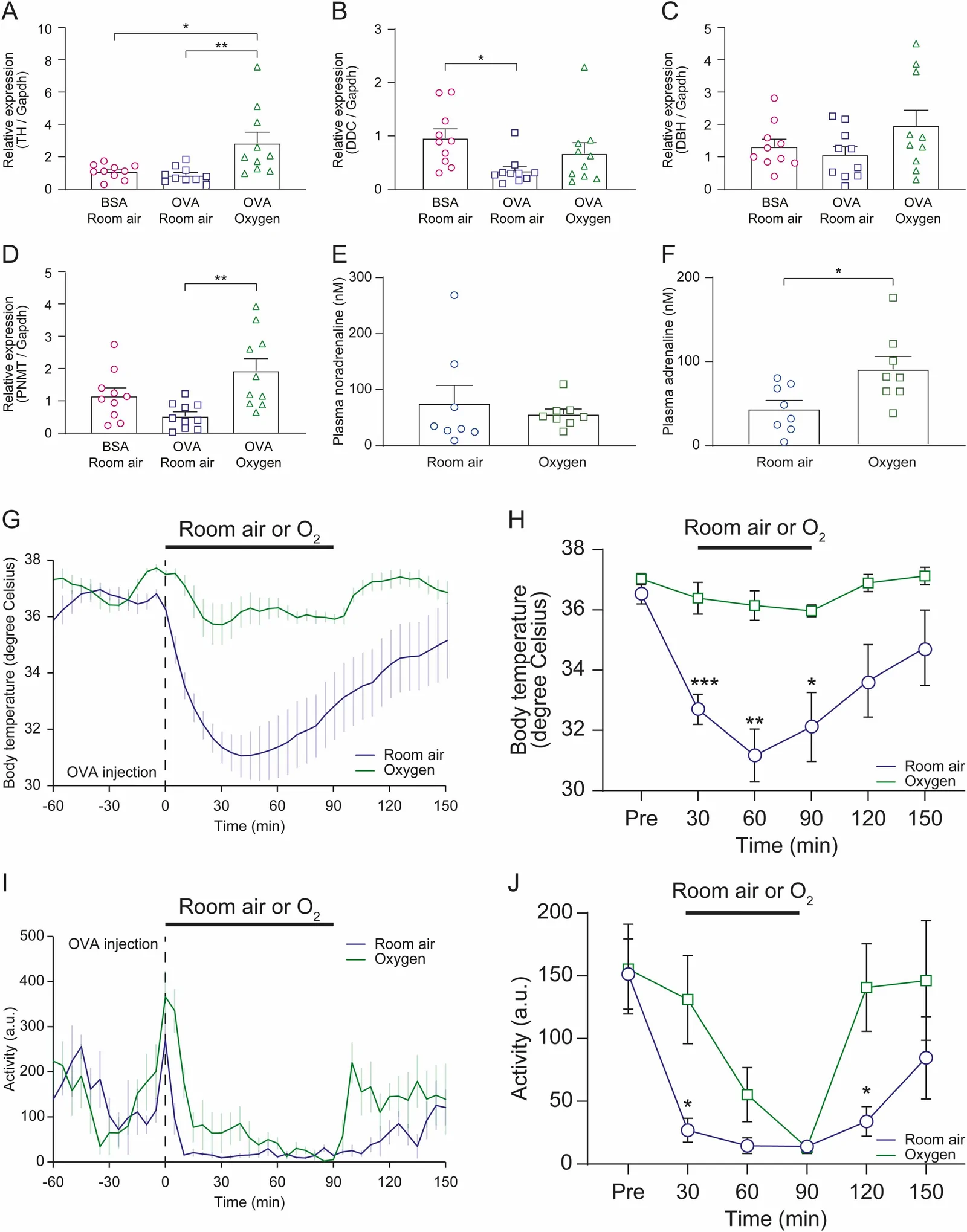
Oxygen supplementation partially restores adrenal adrenergic function and systemic homeostasis during anaphylaxis (A-D) Quantitative PCR analysis of adrenal catecholamine biosynthetic enzyme expression following OVA-induced anaphylaxis under room air or 100% oxygen conditions, including tyrosine hydroxylase (TH; A), dopa decarboxylase (DDC; B), dopamine β-hydroxylase (DBH; C), and phenylethanolamine N-methyltransferase (PNMT; D) (n = 10 per group). Data are presented as mean ± SEM. *P < 0.05 and **P < 0.01 by Tukey’s multiple comparisons test. (E-F) Quantitative analysis of plasma noradrenaline (E) and adrenaline (F) concentrations measured following OVA administration under room air or oxygen supplementation conditions (n = 8 per group). Data are presented as mean ± SEM. *P < 0.05 by unpaired *t*-test. (G-H) Time-course (G) and quantitative analysis (H) of body temperature following OVA administration under room air or oxygen supplementation conditions (n = 5 per group). Data are presented as mean ± SEM. *P < 0.05, **P < 0.01, and ***P < 0.001 versus room air by Tukey’s multiple comparisons test. (I-J) Time-course (I) and quantitative analysis (J) of locomotor activity following OVA administration under room air or oxygen supplementation conditions (n = 5 per group). Data are presented as mean ± SEM. *P < 0.05 and **P < 0.01 versus room air by Tukey’s multiple comparisons test.

Given the enrichment of hypoxia-related pathways in the adrenal transcriptome, we next examined whether oxygen supplementation could ameliorate adrenal dysfunction and systemic physiological deterioration during anaphylaxis. To this end, mice were maintained under either room air or 100% oxygen immediately after OVA administration, and both molecular and physiological responses were evaluated. We first analyzed adrenal expression of catecholamine biosynthetic enzymes. Oxygen supplementation significantly restored the expression of TH and PNMT compared with OVA-challenged mice maintained under room air conditions (Figs. 7A and 7D). In contrast, expression levels of DDC and DBH were not significantly altered by oxygen treatment (Figs. 7B and 7C).

We next evaluated circulating catecholamine levels. Plasma noradrenaline concentrations were not significantly different between room air and oxygen-treated groups (Fig. 7E). However, plasma adrenaline levels were significantly increased in oxygen-treated mice compared with animals maintained under room air conditions (Fig. 7F). Consistent with earlier observations, OVA challenge under room air conditions induced marked hypothermia (Figs. 7G and 7H). In contrast, oxygen-treated mice exhibited significantly attenuated decreases in body temperature, with partial recovery observed throughout the recording period. Similarly, locomotor activity was markedly reduced under room air conditions, whereas oxygen supplementation partially preserved activity levels during anaphylaxis (Figs. 7I and 7J).

## 4 Discussion

The major findings of this study are as follows. 1) The vagal afferent–C1 neuron–adrenal sympathetic nerve pathway remains functionally preserved during food allergy-induced anaphylaxis, and disruption of vagal afferent signaling or adrenal sympathetic innervation exacerbated systemic physiological deterioration and reduced survival. 2) Despite preserved neural activation and elevated plasma catecholamine levels, food allergy-induced anaphylaxis is associated with impaired adrenal adrenaline biosynthetic capacity, characterized by selective downregulation of PNMT, the enzyme responsible for the conversion of noradrenaline to adrenaline. 3) Adrenal transcriptomic analysis revealed enrichment of hypoxia-related pathways, and oxygen supplementation partially restored PNMT expression, plasma adrenaline levels, body temperature, and locomotor activity.

Here, we demonstrate that the vagal afferent–medullary C1 neuron–adrenal sympathetic efferent (AdSNA) pathway remains functionally preserved during systemic anaphylactic shock evoked by food allergens. Electrical stimulation of vagal afferents and optogenetic activation of medullary C1 neurons continued to evoke robust, short-latency adrenal sympathetic responses even after ovalbumin (OVA)-induced anaphylaxis, indicating preserved excitability across the entire reflex arc, from peripheral sensory afferents to central autonomic integrators and adrenal sympathetic efferents. These findings are consistent with and extend previous studies in rats demonstrating selective activation of adrenal and renal sympathetic outflows during anaphylactic shock, rather than generalized sympathetic activation across all autonomic branches [13]. In parallel, previous studies using OVA-induced food allergy models demonstrated that vagal nerve stimulation significantly reduced diarrhea severity, intestinal mast cell accumulation and activation, serum IgE levels, Th2 cytokine production (IL-4, IL-5, and IL-13), and thymic stromal lymphopoietin (TSLP) expression [14]. Similarly, central vagal activation induced by 2-deoxy-D-glucose (2-DG), as well as pharmacological activation of nicotinic acetylcholine receptor signaling using nicotine or the α7 nicotinic receptor agonist GTS-21, suppressed allergic diarrhea and intestinal mast cell hyperplasia, effects that were abolished by nicotinic receptor blockade [15]. Together, these findings support the concept that vagal-autonomic pathways remain functionally recruitable during food allergy-induced anaphylaxis and may contribute not only to cardiovascular homeostasis, but also to the suppression of allergic and inflammatory responses. Importantly, however, this preserved neural activation occurred despite marked systemic physiological deterioration and impaired adrenal adrenergic biosynthetic capacity, suggesting that failure of endogenous compensation during anaphylactic shock may arise not from defective autonomic signaling itself, but from dysfunction within the adrenal medulla.

Despite the functional preservation of autonomic circuitry during anaphylactic episodes, physiological recovery appears to depend critically on the functional integrity of afferent vagal signaling. Our data demonstrate that selective subdiaphragmatic vagal afferent denervation markedly impairs protective autonomic reflexes. Vagal afferent deafferented mice exhibited a more rapid and pronounced decline in core body temperature, along with significantly reduced survival rates compared to sham-operated controls. Previous studies have shown that electrical stimulation of the vagus nerve provides protection against food allergy–induced anaphylactic symptoms [14], and we have previously reported that selective stimulation of vagal afferents elicits anti-inflammatory effects through activation of medullary C1 neurons [11], [16]. Furthermore, anaphylactic reactions have been shown to robustly activate vagal afferent neurons [17], supporting the notion that afferent vagal input is crucial for mounting an effective compensatory response. In contrast, an earlier report found no significant impact of vagotomy on anaphylaxis severity [14]. However, those studies typically employed complete vagotomy, disrupting both afferent and efferent pathways, and often included additional surgical interventions such as pyloroplasty, which may confound physiological interpretation. In the present study, we selectively targeted the afferent limb of the vagus nerve, enabling a more precise evaluation of sensory-to-central autonomic signaling. Taken together, these findings underscore the essential role of vagal afferent input in initiating central autonomic responses that ultimately drive sympathetic outflow to peripheral effectors such as the adrenal medulla.

Parallel to the importance of vagal afferent signaling, the adrenal sympathetic efferent pathway also plays a critical role in systemic defense against anaphylaxis. Mice subjected to bilateral adrenal sympathetic denervation exhibited significantly lower plasma catecholamine levels, particularly adrenaline, compared to controls. This blunted neuroendocrine response was accompanied by severe hypothermia and increased mortality. A previous report has shown that β₂-adrenoceptor blockade exacerbates anaphylactic symptoms [18], [19], underscoring the importance of adrenergic signaling in mitigating allergic responses. Classical immunological studies demonstrated that adrenalectomy enhances experimental hypersensitivity reactions such as anaphylaxis, precipitin responses, and the Arthus phenomenon [20], [21], and increases susceptibility to systemic anaphylactic shock in rats and mice [21], [22]. Furthermore, the AdSNA increases during anaphylactic hypotension [13]. These findings confirm that adrenal sympathetic nerves are essential for mobilizing adrenal medullary catecholamine release.

Interestingly, transcriptional analysis of the adrenal gland revealed a selective downregulation of phenylethanolamine N-methyltransferase (PNMT), the enzyme responsible for catalyzing the final step in adrenaline synthesis by converting noradrenaline to adrenaline. Both RNA-sequencing and quantitative PCR validation confirmed that, unlike upstream catecholamine biosynthetic enzymes such as tyrosine hydroxylase (TH), dopa decarboxylase (DDC), and dopamine β-hydroxylase (DBH), PNMT expression was significantly suppressed in OVA-challenged mice. These findings indicate a potential bottleneck in adrenal catecholamine biosynthesis at the terminal enzymatic step, which may limit sustained adrenaline production during systemic inflammatory stress. Under various physiological stress conditions, adrenal catecholamine biosynthetic enzymes such as TH, DBH, and PNMT are typically upregulated at both the mRNA and protein levels to support increased adrenergic demand [23], [24], [25], [26]. Anaphylaxis likewise represents a profound systemic stress response requiring rapid and sustained sympathoadrenal compensation. Consistent with this concept, plasma adrenaline concentrations were significantly elevated following OVA challenge in the present study. However, plasma catecholamines were evaluated only at a single post-anaphylactic time point, and therefore do not provide information regarding the long-term sustainability of adrenal adrenergic output. If adrenaline biosynthesis is progressively constrained by reduced PNMT expression and impaired enzymatic capacity, endogenous adrenal compensation may become insufficient during the later phases of anaphylactic shock. In addition, previous studies of allergic airway inflammation reported no major alterations in TH expression within sympathetic ganglia, including the superior cervical and stellate ganglia [27], suggesting that generalized impairment of sympathetic catecholaminergic machinery is unlikely. Therefore, the adrenergic dysfunction observed during anaphylaxis may arise predominantly from impaired adrenal medullary adrenaline synthesis rather than from defective catecholamine production within sympathetic nerve terminals. Because adrenal tissues were collected 60 min after OVA administration, the observed changes most likely represent an early transcriptional response to anaphylaxis. Although protein abundance and enzymatic activity were not assessed in the present study, these early transcriptional alterations may contribute to subsequent impairment of adrenal adrenaline biosynthetic capacity during the later stages of anaphylaxis.

Furthermore, pathway enrichment analysis revealed significant activation of hypoxia-related gene programs in the adrenal gland during anaphylaxis, suggesting that adrenal medullary hypoxic stress may contribute to impaired adrenal adrenaline biosynthetic capacity. Importantly, this refers to reduced adrenal adrenaline biosynthetic capacity rather than impaired catecholamine release, because plasma noradrenaline and adrenaline concentrations remained elevated during anaphylaxis. Consistent with this interpretation, oxygen supplementation partially restored PNMT expression and increased circulating adrenaline levels in OVA-challenged mice. Importantly, oxygen treatment also attenuated hypothermia and partially preserved locomotor activity, indicating improved systemic physiological homeostasis. In contrast, plasma noradrenaline levels were not significantly altered by oxygen supplementation, supporting the notion that hypoxia selectively affects adrenal adrenaline biosynthesis rather than global sympathetic activation. Because arterial PCO₂ remained unchanged after OVA administration, severe alveolar hypoventilation was unlikely, suggesting that the hypoxia-related transcriptomic changes in the adrenal gland may primarily reflect impaired tissue perfusion during systemic anaphylaxis. Previous studies have demonstrated that HIF-2α suppresses PNMT expression and reduces adrenal adrenaline synthesis [28], whereas HIF-1α contributes to the maintenance of catecholamine biosynthesis under hypoxic stress through regulation of TH and PNMT transcription [29]. However, despite enrichment of hypoxia-related pathways in the present RNA-seq analysis, we did not detect significant changes in HIF-1α or HIF-2α expression in adrenal tissues following OVA-induced anaphylaxis. In addition, earlier studies in animals exposed to severe hypoxia reported an initial depletion of adrenal catecholamine content followed by recovery associated with enhanced catecholamine synthesis [30]. Together, these findings suggest that hypoxia-associated adrenal dysfunction during anaphylaxis may involve dynamic and temporally regulated mechanisms that functionally limit endogenous adrenergic compensation during severe allergic shock.

As a limitation of the present study, plasma catecholamine levels were measured only at a single time point after anaphylaxis, precluding assessment of the temporal dynamics of adrenal adrenergic compensation. Although vagal afferent denervated mice showed impaired physiological recovery, plasma adrenaline levels were comparable to those of vagal-intact mice at 60 min after OVA challenge. This may reflect a functional ceiling of adrenal adrenaline release or progressive impairment of adrenal biosynthetic capacity during prolonged anaphylaxis. Future studies examining temporal changes in adrenal catecholamine synthesis and release are warranted.

Although arterial hypotension typically activates the arterial baroreflex and elicits reflex tachycardia, severe anaphylaxis can paradoxically be accompanied by bradycardia [31]. In the present study, OVA-IgE/Jcl mice exhibited profound hypotension, marked hypothermia, and metabolic acidosis, indicating a relatively severe anaphylactic phenotype. Under these conditions, vagally mediated cardiodepressor reflexes, direct actions of inflammatory mediators on the heart, and strain-dependent differences may outweigh baroreflex-mediated tachycardia [32]. Importantly, preservation of the vagal afferent–C1 neuron–adrenal sympathetic pathway does not necessarily imply preservation of all cardiovascular reflexes, because regulation of adrenal sympathetic outflow and cardiac chronotropic responses is mediated by partially distinct central autonomic circuits [33]. Thus, the observed bradycardia might not be inconsistent with the preserved adrenal sympathetic reflex demonstrated in the present study.

Furthermore, adrenal sympathetic nerve recordings were performed under urethane anesthesia, which may influence neuroendocrine responses [34]. Nevertheless, baseline AdSNA increased following OVA administration even under urethane anesthesia, indicating preservation of an OVA-induced autonomic response. These experiments were designed to evaluate the integrity of the vagal afferent–C1 neuron–adrenal sympathetic pathway by comparing reflex-evoked responses before and after OVA administration within the same animal, rather than the absolute magnitude of sympathetic activation. The major physiological and molecular findings were obtained from conscious or lightly anesthetized animals.

In conclusion, the present study demonstrates that food allergy-induced anaphylaxis preserves the vagal afferent–C1 neuron–adrenal sympathetic pathway but impairs adrenal adrenaline biosynthetic capacity. Oxygen supplementation partially restored adrenal adrenaline biosynthesis and improved systemic physiological outcomes. These findings suggest that adrenal medullary dysfunction contributes to impaired endogenous adrenergic compensation during anaphylactic shock.

## Authors' contributions

Chikara Abe was responsible for study planning. Marina Khatun, Nishat Akther, Sajjad Hossain, Sakurako Asano, Risa Shimane, Nanaka Tameike, Mamoru Tanida, Yusaku Iwasaki, Yuta Masuda, and Chikara Abe conducted the experiments. All authors checked and revised the manuscript.

## CRediT authorship contribution statement

**Sakurako Asano:** Writing – review & editing, Investigation. **Sajjad Hossain:** Investigation. **Nishat Akther:** Writing – review & editing, Investigation, Formal analysis, Data curation. **Chikara Abe:** Writing – review & editing, Writing – original draft, Visualization, Validation, Supervision, Project administration, Methodology, Investigation, Funding acquisition, Formal analysis, Data curation, Conceptualization. **Marina Khatun:** Writing – review & editing, Investigation, Formal analysis, Data curation. **Yuta Masuda:** Writing – review & editing, Methodology, Investigation, Formal analysis, Data curation. **Yusaku Iwasaki:** Writing – review & editing, Investigation. **Mamoru Tanida:** Writing – review & editing, Investigation. **Yukari Takeda:** Writing – review & editing. **Kakeru Shimizu:** Writing – review & editing, Data curation. **Nanaka Tameike:** Writing – review & editing, Investigation. **Risa Shimane:** Writing – review & editing, Investigation.

## Consent for publication

Not applicable.

## Ethics declaration

This study was conducted in accordance with the ARRIVE (Animal Research: Reporting of In Vivo Experiments) guidelines. The study was approved by the Animal Research Committees of the University of Fukui (R08013) and Gifu University (AG-P-N-20230026).

## Declaration of Generative AI and AI-assisted technologies in the writing process

OpenAI ChatGPT (GPT-5.5) was used solely to assist with English language editing and improve readability of the manuscript.

## Funding

This study was supported by the Japan Science and Technology Agency FOREST (Fusion Oriented Research for disruptive Science and Technology) program (JPMJFR234G to C.A.), the Japan Society for the Promotion of Science Grant-in-Aid for Scientific Research (B) (23K27997 to C.A.), the Japan Society for the Promotion of Science Grant-in-Aid for Challenging Exploratory Research (22K19617 to C.A.) and the Nipponham Foundation for the Future of Food (CA).

## Declaration of Competing Interest

The authors declare that they have no known competing financial interests or personal relationships that could have appeared to influence the work reported in this paper.
